# Expansion of a Specific Plasmodium falciparum PfMDR1 Haplotype in Southeast Asia with Increased Substrate Transport

**DOI:** 10.1128/mBio.02093-20

**Published:** 2020-12-01

**Authors:** Carla Calçada, Miguel Silva, Vitória Baptista, Vandana Thathy, Rita Silva-Pedrosa, Diana Granja, Pedro Eduardo Ferreira, José Pedro Gil, David A. Fidock, Maria Isabel Veiga

**Affiliations:** a Life and Health Sciences Research Institute (ICVS), School of Medicine, University of Minho, Campus Gualtar, Braga, Portugal; b ICVS/3B’s - PT Government Associate Laboratory, Braga/Guimarães, Portugal; c Center for Neuroscience and Cell Biology (CNC), University of Coimbra, Coimbra, Portugal; d Microelectromechanical Systems Research Unit (CMEMS-UMinho), University of Minho, Guimarães, Portugal; e Department of Microbiology and Immunology, Columbia University Irving Medical Center, New York, New York, USA; f Division of Infectious Diseases, Department of Medicine, Columbia University Irving Medical Center, New York, New York, USA; g Centre of Biological Engineering (CEB), Department of Biological Engineering, University of Minho, Braga, Portugal; h Stockholm Malaria Center, Department of Microbiology and Tumour Cell Biology, Karolinska Institutet, Stockholm, Sweden; Universitaetsklinikum Heidelberg; National Institute of Allergy and Infectious Diseases

**Keywords:** malaria, *Plasmodium falciparum*, *pfmdr1*, antimalarial drug resistance, copy number variation, Y184F mutation

## Abstract

Artemisinin-based combination therapies (ACTs) have been vital in reducing malaria mortality rates since the 2000s. Their efficacy, however, is threatened by the emergence and spread of artemisinin resistance in Southeast Asia. The Plasmodium falciparum multidrug resistance protein 1 (PfMDR1) transporter plays a central role in parasite resistance to ACT partner drugs through gene copy number variations (CNV) and/or single nucleotide polymorphisms (SNPs). Using genomic epidemiology, we show that multiple *pfmdr1* copies encoding the N86 and 184F haplotype are prevalent across Southeast Asia. Applying genome editing tools on the Southeast Asian Dd2 strain and using a surrogate assay to measure transporter activity in infected red blood cells, we demonstrate that parasites harboring multicopy N86/184F PfMDR1 have a higher Fluo-4 transport capacity compared with those expressing the wild-type N86/Y184 haplotype. Multicopy N86/184F PfMDR1 is also associated with decreased parasite susceptibility to lumefantrine. These findings provide evidence of the geographic selection and expansion of specific multicopy PfMDR1 haplotypes associated with multidrug resistance in Southeast Asia.

## Abstract (precis)

Global efforts to eliminate malaria depend on the continued success of artemisinin-based combination therapies (ACTs) that target *Plasmodium* asexual blood-stage parasites. Resistance to ACTs, however, has emerged, creating the need to define the underlying mechanisms. Mutations in the P. falciparum multidrug resistance protein 1 (PfMDR1) transporter constitute an important determinant of resistance. Applying gene editing tools combined with an analysis of a public database containing thousands of parasite genomes, we show geographic selection and expansion of a *pfmdr1* gene amplification encoding the N86/184F haplotype in Southeast Asia. Parasites expressing this PfMDR1 variant possess a higher transport capacity that modulates their responses to antimalarials. These data could help tailor and optimize antimalarial drug usage in different regions where malaria is endemic by taking into account the regional prevalence of *pfmdr1* polymorphisms.

## INTRODUCTION

In 2018, an estimated 228 million cases of malaria, predominantly in sub-Saharan Africa, resulted in 405,000 deaths worldwide, mostly in children less than five years old. Plasmodium falciparum is responsible for most of the global malaria burden. Over the past 2 decades, impressive gains have been made in the reduction of global malaria morbidity and mortality rates, which stood at an estimated one million deaths per year in the early 2000s (1). The widespread adoption of artemisinin (ART)-based combination therapies (ACTs) and increased population-level coverage of insecticide-treated bed nets to protect against infective *Anopheles* mosquito vectors have been important contributors in the fight against malaria (2, 3). Unfortunately, the emergence and spread of ART- and ACT partner drug-resistant P. falciparum lineages in the Greater Mekong subregion of Southeast Asia (4–9) and in South America (10, 11) during the past decade pose a significant threat to malaria control and elimination. Efforts are under way to contain the spread of ART resistance into Africa and India (12–14) where it is predicted to have devastating results (15), as happened earlier with the emergence and global spread of chloroquine (CQ) resistance (8, 16, 17).

One important mediator involved in multidrug resistance to ACTs is the ATP-binding cassette (ABC) transporter P. falciparum multidrug resistance protein 1 (PfMDR1). PfMDR1 is localized in the membrane of the digestive vacuole (DV) of the parasite and possesses the ability to influx antimalarial drugs toward the lumen of this organelle (18–20). Transport studies using heterologous expression systems have shown that PfMDR1 variants have the ability to modulate the transport of the antimalarial drugs halofantrine and quinine (QN) (21). A surrogate assay of PfMDR1 activity using the fluorochrome Fluo-4 revealed its subcellular distribution and showed that PfMDR1-mediated solute import into the parasite DV is modulated by at least three C-terminal codon variants (S1034C, N1042D, and D1246Y) (22, 23). The *pfmdr1* N-terminal allelic variants N86Y and Y184F have also been implicated in multidrug-resistant phenotypes. In Africa and Asia, the N86 allele is relevant for *in vivo* and *in vitro* parasite antimalarial responses against aryl-amino alcohols such as mefloquine (MFQ) (24, 25) and lumefantrine (LMF) (26–28). In clinical trials of artemether-LMF or artesunate plus amodiaquine (ADQ), an increased risk of posttreatment recrudescent P. falciparum infections was associated with parasites harboring the N86 wild-type or 86Y mutant allele, respectively (29). These *in vivo* observations have been supported by recent allelic exchange approaches (30). Mutations in *pfmdr1* have a drug-specific resistance contribution being “pro and con” depending on the antimalarial. Compared to *pfmdr1* N86Y, the impact of the Y184F mutation on parasite responses to ACTs remains less studied (31). This mutation is one of the five canonical PfMDR1 mutations that was earlier considered to be associated with chloroquine resistance (32) and is now widespread in Southeast Asia and Africa (33–36). The capacity of 184F to influence parasite antimalarial responses is thought to be related to alterations in protein structure through allosteric effects that reduce drug binding or alter transport kinetics (20).*pfmdr1* copy number variation (CNV) is another important factor that impacts parasite susceptibility to several antimalarial drugs (18). P. falciparum parasites harboring *pfmdr1* amplifications are widely distributed in South America (37) and Southeast Asia (24, 25). Amplification of *pfmdr1* is associated with an increased risk of treatment failure with therapies combining an aryl-amino alcohol such as MFQ or LMF with an ART derivative (25, 38, 39).

Using data from the updated MalariaGEN Plasmodium falciparum Community Project (40), we explored the prevalence and distribution of *pfmdr1* copy numbers encoding specific haplotypes. We show that *pfmdr1* gene amplification is especially common in Southeast Asia, with amplifications being selected along with the N86 and 184F alleles. Applying genome editing tools on the 86Y- and Y184-harboring Southeast Asian Dd2 strain, we demonstrate that the N86/184F haplotype in the context of amplified *pfmdr1* imparts a higher transport capacity that directly affects parasite antimalarial responses.

## RESULTS

### Temporal distribution of PfMDR1 haplotypes and copy number variants in Southeast Asia.

We determined the worldwide frequencies of *pfmdr1* N86Y and Y184F mutations and *pfmdr1* copy number using a collection of 7,113 parasite genomes from the MalariaGEN P. falciparum Community Project version 6 (https://www.malariagen.net/resource/26) (40). Genomic data were available from 73 countries where malaria is endemic with information on *pfmdr1* N86Y and Y184F mutations, as well as *pfmdr1* copy number, in a subset of 5,003 samples. From that subset, 650 had *pfmdr1* amplifications. Of these, 53 were excluded from further frequency analysis due to the presence of multiple alleles at codon positions 86 and/or 184, indicating the presence of mixed infections (see Table S1 in the supplemental material).*pfmdr1* allele frequency data were extracted from the MalariaGEN Community Project during the period from 2002 to 2015, revealing that the *pfmdr1* N86Y mutation frequency has remained relatively constant in Southeast Asia, with the N86 allele nearly fixed at a frequency of 96 to 98% (N86 allele frequency, 98% between 2002 and 2007, 98% between 2008 and 2010, 99% between 2011 and 2013, and 96% between 2014 and 2015). In Africa, the N86 allele frequency has increased, whereas 86Y has decreased over the same time period (see Fig. S1 in the supplemental material). In contrast to the fixation of the N86 allele in Southeast Asia, the frequency of the 184F variant has increased within the same time frame (21% between 2002 and 2007, 30% between 2008 and 2010, 40% between 2011 and 2013, and 59% between 2014 and 2015), leading to the selection of the N86/184F haplotype in Southeast Asia between 2002 and 2015 (Fig. S1).

10.1128/mBio.02093-20.1 FIG S1 Worldwide frequencies of *pfmdr1* N86Y and Y184F mutations. Genetic analysis used data generated by the recent updated MalariaGEN Plasmodium falciparum Community Project (version 6; https://www.malariagen.net/resource/26) (40) and comprised genomes with information on *pfmdr1* gene copy number, SNPs, and showed no evidence of mixed infections. Bars represent the allelic frequency calculated for each allele or combination of alleles and are stratified into four intervals of time (2002 to 2007, 2008 to 2010, 2011 to 2013, and 2014 to 2015). Allele frequencies were determined for the worldwide sample set and were then stratified into three regional categories: Southeast Asia, South America, and Africa. Error bars are 95% confidence intervals. Time intervals without information or below 10 genomes are not represented. Download FIG S1, TIF file, 0.7 MB. Copyright © 2020 Calçada et al. 2020 Calçada et al. This content is distributed under the terms of the Creative Commons Attribution 4.0 International license.

10.1128/mBio.02093-20.3 TABLE S1 Prevalence of PfMDR1 haplotypes at amino acid positions 86 and184 and copy number variation. Genetic analysis used data from the MalariaGEN P. falciparum Community Project (version 6) (https://www.malariagen.net/resource/26) (40). Download Table S1, XLSX file, 0.02 MB. Copyright © 2020 Calçada et al. 2020 Calçada et al. This content is distributed under the terms of the Creative Commons Attribution 4.0 International license.

**FIG 1:**
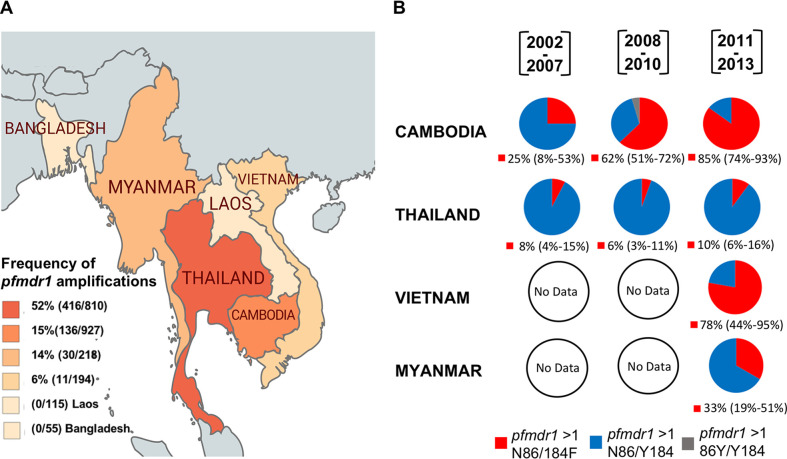
Geographical distribution of amplified *pfmdr1* gene copies and haplotype selection in Southeast Asia. Genetic analysis used data generated by the recently updated MalariaGEN Plasmodium falciparum Community Project (version 6; https://www.malariagen.net/resource/26) (40), comprising genomes with information on *pfmdr1* copy number, single nucleotide polymorphisms (SNPs), and no evidence of mixed infections. (A) Country shadings show the frequency of genomes with amplified *pfmdr1* copies (number of amplified *pfmdr1* copies/total number of genomes) collected between 2002 and 2015. (B) Pie charts represent different PfMDR1 haplotypes present in genomes containing multiple *pfmdr1* gene copies (per country data are shown in Table S1 in the supplemental material) and stratified into three intervals of time (2002 to 2007, 2008 to 2010, and 2011 to 2013). No samples were available for the years 2014 and 2015. The N86/184F haplotype frequency is shown with 95% confidence intervals in parentheses below each pie chart. Empty pie charts represent time periods in countries where no data were available.

The MalariaGEN Community Project data also showed that *pfmdr1* amplifications are the most prevalent in Southeast Asia, with only one African isolate reported in 2009 from Ghana harboring two *pfmdr1* copies encoding a N86/Y184 haplotype (Table S1). In Southeast Asia, infections with P. falciparum isolates harboring *pfmdr1* amplifications were most prevalent in Thailand (52%), followed by Cambodia, Myanmar, and Vietnam (15%, 14%, and 6%, respectively) (Fig. 1A). In Cambodia, the frequency of isolates with *pfmdr1* amplifications decreased progressively from 48% (12/25 isolates) during 2002 to 2007 to 25.6% (69/269 isolates) during 2008 to 2010 and 9.6% (55/572 isolates) between 2011 and 2013) (41, 42). In contrast to Cambodia, the frequency of isolates with *pfmdr1* gene amplifications in Thailand showed an increasing trend over the same period (45% [104/229] during 2002 to 2007, 47% [144/306] during 2008 to 2010, and 61% [168/275] during 2011 to 2013). Genomes containing *pfmdr1* amplifications encoding the N86 allele predominated (97.8%), with only three isolates identified in Cambodia carrying amplified copies of the *pfmdr1* 86Y variant (3/136 genomes) (Fig. 1B and Table S1).

In Cambodia, the 184F allele frequency in isolates also carrying multiple copies of the *pfmdr1* N86 allele has increased (25% [3/12] between 2002 and 2007% to 62% [43/69] between 2008 and 2010 and 85% [47/55] between 2011 and 2013). In contrast, in Thailand, the 184F allele frequency has remained stable in isolates containing multicopy *pfmdr1* over the years (8% [8/104] between 2002 and 2007% to 6% [8/144] between 2008 and 2010 and 10% [17/168] between 2011 and 2013). Limited or no genome data were available from Vietnam and Myanmar between 2002 and 2010, precluding the analysis of haplotype selection over time in these countries (Fig. 1B and Table S1).

Together, these results show that *pfmdr1* amplification is especially common in Southeast Asia, with amplifications encoding the N86 and 184F residues being selected.

### Amplified *pfmdr1*-edited parasites at codons 86 and 184.

We developed a targeted gene editing approach to study the impact of haplotype-specific increases in *pfmdr1* copy number on P. falciparum. Parasite lines derived from Southeast Asian P. falciparum isolates from the 1980s, like Dd2, characteristically contain the 86Y allele. To investigate the advantage of the predominant genotypes observed in Southeast Asia described above (*pfmdr1* amplifications containing haplotypes N86/184F or N86/Y184), we performed gene editing in all *pfmdr1* gene copies present in Dd2 parasites. Our Dd2 line represents a MFQ-resistant clone carrying four *pfmdr1* copies isolated from the W2mef line, originally derived from the Southeast Asian CQ-resistant parasite strain W2 (harboring a single copy of *pfmdr1*) upon extended culture with MFQ for 96 weeks (43). Dd2 and W2 parasites express the PfMDR1 86Y/Y184 haplotype and the CQ resistance-conferring CVIET haplotype of the P. falciparum chloroquine resistance transporter (PfCRT) (Table 1) (44, 45).

**TABLE 1:** *pfmdr1* and *pfcrt* polymorphisms of gene-edited and parental P. falciparum laboratory strains

Parasite	Plasmid	*pfmdr1* polymorphisms						*pfcrt* polymorphisms				
		CNV	86	184	1034	1042	1246	72	73	74	75	76
NF^Dd2^	p*mdr1*^NF^^*a*^	4	N	F	S	N	D	C	V	I	E	T
NY^Dd2^	p*mdr1*^NY^^*a*^	4	N	Y	S	N	D	C	V	I	E	T
Dd2		4	Y	Y	S	N	D	C	V	I	E	T
W2		1	Y	Y	S	N	D	C	V	I	E	T

a Plasmids were previously constructed (30). CNV, copy number variation.

**FIG 2:**
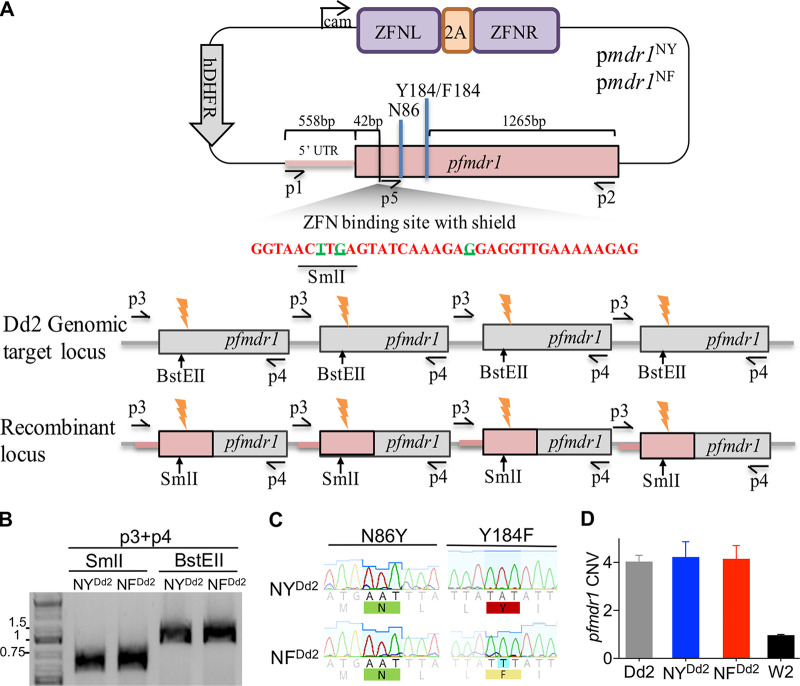
Schematic representation of the ZFN-based *pfmdr1* editing strategy. (A) The plasmids p*mdr1*^NY^ and p*mdr1*^NF^ (30) harbor a pair of *pfmdr1*-specific ZFNs (ZFN left [ZFN L] and ZFN right [ZFN R]) separated by a 2A skip peptide that drives their polycistronic expression from the calmodulin promoter (cam), and the human dhfr (hdhfr) selectable marker. The ZFNs induce a double-strand break (orange thunderbolt) 42 bp downstream of the *pfmdr1* start codon, which is repaired by homologous recombination with the 2.4-kb *pfmdr1* donor sequence present on the plasmid. The *pfmdr1* donor sequence from p*mdr1*^NY^ and p*mdr1*^NF^ plasmids contains the N86/Y184 or N86/184F genotypes, respectively, and carries three synonymous mutations (nucleotides colored in green) at the ZFN binding site to prevent ZFNs from cleaving the plasmids or the *pfmdr1*-edited sequences. Two of the three synonymous mutations create a SmlI restriction site and abolish a BstEII restriction site present in the nonedited (genomic) template. (B) PCR-RFLP genotyping confirming the presence of *pfmdr1*-edited lines NY^Dd2^ and NF^Dd2^. The silent mutations introduced into the donor sequence created a SmlI restriction site, which was used to screen PCR products (yielding a 1.3-kb band with P3 and P4 primers, which formed a doublet at 667/669 bp in edited parasites upon SmlI digestion). BstEII cuts the P3 and P4 PCR product in nonedited parasites and was used as a control; the observed 1.3-kb band was not cut, indicating the presence of correctly edited parasites. (C) Sanger sequencing chromatograms of DNA samples from the NY^Dd2^ and NF^Dd2^ edited lines at the *pfmdr1* locus, confirming correct editing at codons 86 and 184. (D) Plot showing means plus standard errors of the means (SEM) (error bars) from three independent assays of *pfmdr1* copy number, analyzed through quantitative PCR using β-tubulin as a control gene and the 3D7 strain as a calibrator with a single *pfmdr1* copy.

Using the previously described zinc-finger nuclease (ZFN) strategy to engineer *pfmdr1* mutations at codons 86 and 184, we successfully generated the Dd2 edited lines, NF^Dd2^ and NY^Dd2^, each maintaining four copies of *pfmdr1* encoding the N86/184F and N86/Y184 haplotypes, respectively (Table 1 and Fig. 2A and B). These edited lines represent the two major geographic variants of *pfmdr1* present in Southeast Asia and include gene amplifications. The presence of pure N86/184F and N86/Y184 haplotypes in NF^Dd2^ and NY^Dd2^ lines, respectively, was confirmed by Sanger sequencing (Fig. 2C). Quantitative PCR analysis demonstrated that the edited NF^Dd2^ and NY^Dd2^ lines maintained four copies of *pfmdr1* as present in the unedited Dd2 parental strain (Fig. 2D). Attempts to engineer parasites expressing the 86Y/184F haplotype failed. We verified that this haplotype combination was not found among the 597 parasite genomes harboring amplified copies of *pfmdr1* in the MalariaGEN Community Project data set (Table S1). These findings suggest that the presence of the 86Y/184F haplotype in amplified *pfmdr1* copies might render the parasites nonviable or might confer a survival disadvantage to the parasite population.

### Fluo-4 transport into the parasite DV is altered by amplified PfMDR1 haplotypes.

We evaluated the influence of *pfmdr1* polymorphisms at codons 86 and 184 on PfMDR1 functional activity by measuring the accumulation of Fluo-4 in the DVs (22) of the gene-edited parasite lines (NF^Dd2^ and NY^Dd2^) compared to the Dd2 parental control strain.

**FIG 3:**
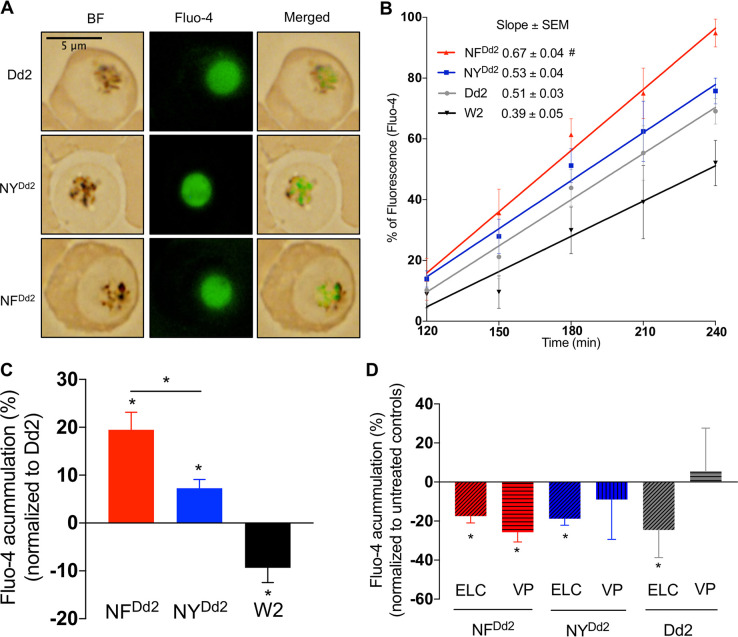
Evaluation of Fluo-4 accumulation. (A) Representative images of Dd2 and *pfmdr1*-edited lines (NY^Dd2^ and NF^Dd2^) stained with Fluo-4 AM (green). BF, Bright field. The bar measures 5 μm. (B) PfMDR1-associated dynamic transport between the strains Dd2, NF^Dd2^, NY^Dd2^, and W2, as determined by linear regression of the Fluo-4 probe signal over time. Data are shown as comparative mean ± SEM (error bars) (as a percentage) from four independent assays. Linear regression and slopes extracted from each equation. # indicates a significant difference in the rate of Fluo-4 accumulation in NF^Dd2^ parasites compared with the rates obtained in NY^Dd2^ and Dd2 parasites (*P* = 0.04 and *P* = 0.007, respectively). (C) Normalized Fluo-4 AM intensities (as a percentage) in all parasite strains measured at 240 min. The fluorescence intensities exhibited by NF^Dd2^ and NY^Dd2^ and the W2 strain were normalized to the parental Dd2 parasite strain (indicated in the graph as the baseline [*y* = 0]). Values below or greater than zero indicate a decrease or increase, respectively, in the percentage of accumulated fluorescence compared with Dd2 parasites. Mann-Whitney U tests were used to assess statistically significant differences between the NF^Dd2^, NY^Dd2^, Dd2, and W2 parasites in panels B and C (*, *P* < 0.05). (D) Normalized Fluo-4 intensities (as a percentage) in the absence (untreated control) and presence of elacridar (ELC, 0.1 μM) and verapamil (VP, 0.8 μM). Results were normalized to untreated controls (indicated in the graph as the baseline [*y* = 0]). Values below or greater than zero indicate a decrease or increase, respectively, in the percentage of accumulated fluorescence compared with untreated controls. The means plus SEM of four independent measurements are shown. Mann-Whitney U tests were used to assess statistically significant differences between untreated and treated parasites (*, *P* < 0.05).

Accumulation of Fluo-4 in the DVs of NF^Dd2^, NY^Dd2^, and Dd2 parasites was observed by fluorescence microscopy and measured by flow cytometry (Fig. 3). Fluo-4 accumulation in the DV of the parasite was detectable after 120 min of incubation, prior to which time the probe was distributed throughout the infected red blood cell (RBC) and parasite cytosol (Fig. S2).

10.1128/mBio.02093-20.2 FIG S2 Accumulation of Fluo-4 in arbitrary units of fluorescence over time measured by flow cytometry. Parasites were incubated with Fluo-4 AM (5 μM) for 240 min at 37°C under a humidified controlled atmosphere recording fluorescence by flow cytometer, every 15 min until 120 min and then every 30 min until 240 min. Prior to flow cytometry, live-cell imaging was performed at all time points described above to visualize when the Fluo-4 probe entered the parasite DV. Images presented are representative of the time points. For the flow cytometry assays, the parasites were also loaded with Mitotracker Deep Red FM 30 min before the incubation ended. The scale bar measures 10 μm. Download FIG S2, TIF file, 0.4 MB. Copyright © 2020 Calçada et al. 2020 Calçada et al. This content is distributed under the terms of the Creative Commons Attribution 4.0 International license.

Time variances in Fluo-4 transport associated with the PfMDR1 N86/184F and N86/Y184 haplotypes in the edited parasite lines were measured by applying a linear regression model from 120 min to 240 min (Fig. 3B). We observed a higher positive slope in the edited NF^Dd2^ parasites that express the 184F variant (slope = 0.67 ± 0.04), compared with NY^Dd2^ (*P* = 0.04) or the Dd2 parental strain (86Y/Y184) (*P* = 0.007) (slope = 0.53 ± 0.04 and 0.51 ± 0.03, respectively), suggesting a nonnegligible influence of amino acid position 184 in PfMDR1 transport capacity (Fig. 3B).

To determine whether there were PfMDR1 haplotype-specific differences in the accumulation of Fluo-4, we measured the Fluo-4 intensity of the *pfmdr1*-edited lines and the W2 comparator strain after normalizing to the fluorescence intensity of Dd2 parasites after 240 min. The results showed that the N86 allele has an important role in the modulation of the PfMDR1 transporter since N86 edited parasites accumulated more Fluo-4 compared with the Dd2 parental strain containing the 86Y allele (*P* = 0.03). Furthermore, edited NF^Dd2^ parasites containing the N86/184F haplotype accumulated more Fluo-4 in their DVs than edited NY^Dd2^ parasites (haplotype N86/Y184) (*P* = 0.03). W2 parasites, which harbor a single genomic copy of the *pfmdr1* gene with the 86Y/Y184 haplotype, accumulated less Fluo-4 (*P* = 0.03) relative to the parental Dd2 control strain that expresses four copies with the same 86Y/Y184 haplotype (Fig. 3C). The influence of *pfmdr1* copy number on the import of Fluo-4 into the parasite DV observed herein confirms previous reports where a parasite strain harboring only one *pfmdr1* copy accumulated less Fluo-4 compared with parasites expressing two copies (31).

To determine whether the changes in Fluo-4 accumulation were related to the PfMDR1 haplotype, we used elacridar (ELC) as a tool, since it is a well-established inhibitor of P-glycoprotein type ABC transporters (46). We measured the fluorescence intensity of Fluo-4 in NF^Dd2^, NY^Dd2^, or Dd2 parasites in the presence of ELC. Data were normalized to the respective untreated controls (NF^Dd2^, NY^Dd2^, or Dd2; indicated in Fig. 3D as the baseline [*y* = 0]). We observed a significantly decreased Fluo-4 accumulation in ELC-treated parasites compared with untreated controls (*P* = 0.03). The addition of verapamil (VP), a calcium ion channel blocker that reverses parasite resistance to CQ, led to a significant reduction of Fluo-4 accumulation only in the edited NF^Dd2^ line compared to untreated NF^Dd2^ (*P* = 0.03), suggesting a possible interplay between the PfMDR1 and PfCRT transporters in 184F-expressing parasites (Fig. 3D). The addition of VP produced a trend toward more Fluo-4 accumulation in NY^Dd2^ and Y184-harboring Dd2 parasites compared with NF^Dd2^. Nevertheless, despite having a different trend, the quite large standard error of the mean (SEM) made these results inconclusive.

### Amplified *pfmdr1* with the N86 allele decreases parasite susceptibility to antimalarials.

**FIG 4:**
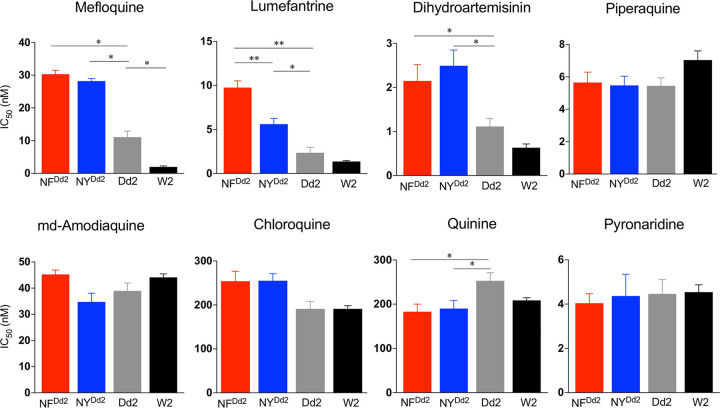
*In vitro* IC_50_ antimalarial response of NF^Dd2^, NY^Dd2^, Dd2, and W2 parasite strains. The antimalarial drugs (mefloquine [MFQ], lumefantrine [LMF], dihydroartemisinin [DHA], piperaquine [PPQ], monodesethyl-amodiaquine [md-ADQ], chloroquine [CQ], quinine [QN], and pyronaridine [PND]) were serially diluted, and after 72 h, the parasitemias were determined by flow cytometry after staining the parasites with Mito Tracker Deep Red FM and SYBR green. IC_50_ values were calculated using nonlinear regression analysis. Data are means plus SEM of at least three independent assays. Mann-Whitney U tests were used to assess statistically differences between Dd2 parental strain and NF^Dd2^, NY^Dd2^, and W2 strains. *, *P* < 0.05; **, *P* < 0.01.

We determined the impact of the N86 allele in the genomic context of multicopy *pfmdr1* on parasite susceptibility to antimalarial drugs. Replacement of the 86Y variant present in Dd2 with N86 in each of the four *pfmdr1* copies (edited parasite line NY^Dd2^) induced a significant increase in the half-maximal *in vitro* growth inhibitory concentration (IC_50_) values for MFQ (2.5-fold, *P* = 0.02) and LMF (2.4-fold, *P* = 0.02), as well as for dihydroartemisinin (DHA) (2.3-fold, *P* = 0.05) (Fig. 4 and Table 2). A reduction in the IC_50_ values for MFQ (6.1-fold, *P* = 0.05) in W2 (a single *pfmdr1* copy strain with the 86Y/Y184 haplotype) compared with Dd2 parasites was observed. These data show a clear role of *pfmdr1* gene amplifications, particularly those encoding the N86 allelic variant, in the multidrug resistance phenotype.

**TABLE 2:** Parasite responses to antimalarials in the presence of verapamil or elacridar^*a*^

Antimalarial drug(s)	NF^Dd2^		NY^Dd2^		Dd2	
	Mean IC_50_ (± SEM)	*P* value	Mean IC_50_ (± SEM)	*P* value	Mean IC_50_ (± SEM)	*P* value
MFQ	30.3 (±1.1)		28.2 (±1.0)		11.1 (±1.8)	
MFQ + VP	7.5 (±0.4)	0.03	5.6 (±0.8)	0.03	4.2 (±0.1)	0.03
MFQ + ELC	10.9 (±0.7)	0.03	11.1 (±1.5)	0.03	7.7 (±0.8)	NS
LMF	9.7 (±0.8)		5.6 (±0.6)		2.3 (±0.6)	
LMF + VP	4.4 (±0.3)	0.01	3.0 (±0.2)	0.008	2.5 (±0.3)	NS
LMF + ELC	6.8 (±1.7)	NS	5.5 (±0.8)	NS	3.6 (±0.4)	NS
DHA	2.1 (±0.4)		2.5 (±0.4)		1.1 (±0.2)	
DHA + VP	1.2 (±0.1)	NS	1.3 (±0.1)	NS	1.0 (±0.1)	NS
DHA + ELC	1.5 (±0.1)	NS	1.8 (±0.09)	NS	1.2 (±0.1)	NS
PPQ	5.7 (±0.6)		5.5 (±0.6)		5.4 (±0.5)	
PPQ + VP	4.8 (±0.9)	NS	4.7 (±0.2)	NS	4.9 (±0.5)	NS
PPQ + ELC	5.9 (±1.2)	NS	5.1 (±0.5)	NS	5.0 (±0.7)	NS
md-ADQ	45.2 (±1.7)		34.7 (±3.4)		38.9 (±3.0)	
md-ADQ + VP	21.7 (±1.0)	0.05	15.0 (±2.8)	0.05	14.3 (±1.2)	0.05
md-ADQ + ELC	39.6 (±3.9)	NS	35.4 (±3.2)	NS	43.3 (±1.4)	NS
CQ	254.1 (±15.8)		255.1 (±22.6)		191.2 (±16.6)	
CQ + VP	45.8 (±2.7)	0.008	47.2 (±2.8)	0.004	37.1 (±2.6)	0.004
CQ + ELC	211.9 (±18.4)	NS	224.7 (±19.2)	NS	169.2 (±6.6)	NS
QN	183.1 (±16.9)		190.0 (±18.1)		252.8 (±18.3)	
QN + VP	65.3 (±22.8)	0.003	60.6 (±33.3)	0.003	111.5 (±3.3)	0.003
QN + ELC	198.1 (±39.6)	NS	211.2 (±57.6)	NS	263.2 (±19.5)	NS
PND	4.1 (±0.4)		4.4 (±0.9)		4.4 (±0.6)	
PND + VP	2.5 (±0.5)	NS	3.4 (±0.2)	NS	3.0 (±0.3)	NS
PND + ELC	2.6 (±0.6)	NS	2.7 (±0.3)	NS	2.6 (±0.2)	0.05

a NF^Dd2^, NY^Dd2^, and Dd2 parasites were incubated with mefloquine (MFQ), lumefantrine (LMF), dihydroartemisinin (DHA), piperaquine (PPQ), monodesethyl-amodiaquine (md-ADQ), chloroquine (CQ), quinine (QN), and pyronaridine (PND) in the presence or absence of verapamil (VP) or elacridar (ELC). When incubated with verapamil or elacridar, the antimalarial drugs used in study were serially diluted in the presence of 0.8 μM VP or 0.1 μM ELC for the 72-h *in vitro* growth inhibition assays. Parasitemias were determined by flow cytometry after staining the parasites with Mito Tracker Deep Red FM and SYBR green. Mean ± SEM IC_50_ values (nanomolar) were derived from three to five independent drug assays. Mann-Whitney U tests were used to assess statistically significant differences between the untreated control parasites (Dd2, NY^Dd2^, and NF^Dd2^) and VP- or ELC-treated parasites. NS, not significant.

In the case of QN, the replacement of 86Y to N86 in multicopy *pfmdr1* resulted in a decrease of IC_50_ values in the NF^Dd2^ and NY^Dd2^ lines (1.6-fold, *P* = 0.02) relative to the parental Dd2 strain.

Our assays revealed no differences in susceptibilities to the quinoline drugs CQ, monodesethyl-ADQ (md-ADQ), piperaquine (PPQ), and pyronaridine (PND) in the context of multicopy *pfmdr1* containing N86.

### The N86/184F PfMDR1 haplotype decreases parasite susceptibility to LMF.

Analysis of the MalariaGEN P. falciparum Community Project data predicted a prevalence of the *pfmdr1* 184F allele in Africa of 58.6% (184F, 1,438/2,454 genomes sampled between 2002 and 2015) and 36% (184F, 858/2,403 between 2002 and 2015) in Southeast Asia, regardless of the number of *pfmdr1* copies (Table S1 and Fig. S1). The frequency of the 184F allele increased worldwide over the years analyzed (Table S1 and Fig. S1). Here, in the context of amplified *pfmdr1* gene copies, we observed up to 1.7-fold (*P* = 0.002) decreased susceptibility to LMF in edited parasites expressing the N86/184F haplotype compared with isogenic parasites expressing the N86/Y184 haplotype (Fig. 4 and Table 2). This suggests a specific effect of the 184F allele in conferring resistance to LMF in parasites that harbor amplified copies of *pfmdr1*.

### PfMDR1-mediated resistance phenotypes are modulated by VP and ELC.

We investigated the potential interplay between PfMDR1 and PfCRT transporters in modulating parasite susceptibility to antimalarials using the PfCRT and P-type ATPase transporter inhibitors VP and ELC, respectively. All the parasite strains used in this study express the CVIET variant at amino acid positions 72 to 76 of PfCRT (Table 1). In the presence of VP, the multicopy *pfmdr1* Dd2 strain and the edited parasites lines NF^Dd2^ and NY^Dd2^ exhibited increased susceptibility to the 4-aminoquinolines CQ and md-ADQ compared with the respective untreated parasites, with 5-fold and 2- to 3-fold changes, respectively. Increases in susceptibility were also obtained with QN and MFQ in the presence of VP compared with untreated parasites, with N86 producing higher fold changes than 86Y (QN, NF^Dd2^ = 2.8-fold, NY^Dd2^ = 3.1-fold, and Dd2 = 2.2-fold; MFQ, NF^Dd2^ = 4.1-fold, NY^Dd2^ = 5.0-fold, and Dd2 = 2.9-fold) (Table 2). The differences in fold changes observed in the presence of VP can be explained by the differential basal levels of susceptibility to QN and MFQ in the absence of VP in NF^Dd2^, NY^Dd2^, and Dd2 parasites (Fig. 4). For LMF, VP sensitized NF^Dd2^ and NY^Dd2^ parasites up to twofold with no effect observed on unedited Dd2 parasites compared with untreated parasites (Table 2).

Comparing the IC_50_ values between the three haplotypes in the presence of VP, a significant increase in QN susceptibility was observed in NF^Dd2^ and NY^Dd2^ parasites compared with the Dd2 parental control (NY^Dd2^ IC_50_ = 60.6 nM versus Dd2 IC_50_ = 111.5 nM, *P* = 0.01; NF^Dd2^ IC_50_ = 65.3 nM versus Dd2 IC_50_ = 111.5 nM, *P* = 0.02). For MFQ and LMF, there was an increase in IC_50_ values for NF^Dd2^ in the presence of VP compared with VP-treated Dd2 parasites (MFQ NF^Dd2^ IC_50_ = 7.5 nM versus Dd2 IC_50_ = 4.2 nM, *P* = 0.005; LMF NF^Dd2^ IC_50_ = 4.4 nM versus Dd2 IC_50_ = 2.5 nM, *P* = 0.01).

In the presence of the P-type ATPase transporter inhibitor ELC, the parasite lines NF^Dd2^ and NY^Dd2^ exhibited 2.8-fold and 2.5-fold increased susceptibility to MFQ, respectively, compared with the corresponding untreated parasites. It is interesting to observe that ELC treatment reverted the MFQ IC_50_ values of NF^Dd2^ and NY^Dd2^ parasites to the same level as that of Dd2 in the absence of ELC, validating the impact of the N86 *pfmdr1* variant. ELC treatment did not significantly impact the susceptibility of Dd2, NY^Dd2^, or NF^Dd2^ parasites to any of the other aryl-amino alcohols tested (i.e., QN and LMF) or the 4-aminoquinolines (CQ and md-ADQ).

## DISCUSSION

The propensity of P. falciparum to develop antimalarial drug resistance underscores the importance of characterizing its genetic basis. The amplification of *pfmdr1* was the first candidate mechanism implicated in CQ resistance (CQR) (47). Although we now know that PfCRT is the primary determinant of CQR (48), *pfmdr1* can modulate the degree of resistance (49, 50). Several other studies have also shown that increased *pfmdr1* copy number decreases the parasite’s susceptibility to other antimalarials such as MFQ, LMF, and ART (25, 38, 39). The worldwide spread of the *pfmdr1* N-terminal 86Y and 184F mutations have also been linked with differential responses to multiple quinoline-based antimalarials and ART, both *in vitro* and *in vivo* (33, 36, 51–54). The conjugation of these two types of polymorphisms (gene copy number and specific sequence haplotypes) could modulate the P. falciparum response to these antimalarials.

Using data from the MalariaGEN P. falciparum Community Project, we determined the worldwide prevalence and temporal changes of *pfmdr1* copy number with specific *pfmdr1* N-terminal mutations. Furthermore, we measured the impact of these polymorphisms on solute kinetics of this transporter. We note that the MalariaGEN data from a given period (2002 to 2015 in our case) relates to samples whose locations were dependent on which partner studies were operative at the time (40). Temporal trends in aggregated data should therefore be interpreted with caution. From 73 countries where malaria is endemic with information on *pfmdr1* amplifications and mutations at amino acid positions 86 and 184, between 2002 and 2015, *pfmdr1* amplification was found almost exclusively in Southeast Asia, where we also observed a near fixation of the N86 allele (Fig. 1; see also Table S1 and Fig. S1 in the supplemental material). Considering the previous strong association of *pfmdr1* amplifications and the N86 allele with treatment failures that included MFQ or LMF (24, 25, 39), the frequencies observed could be explained by the extensive use of artesunate plus MFQ and artemether plus LMF in this region during that time period. Thailand, for example, is the country with the highest frequency of *pfmdr1* amplifications, possibly reflecting the extensive use of artesunate-MFQ as a first-line antimalarial treatment. Its failure during the following years led to changes in treatment policy, with the adoption of dihydroartemisinin (DHA)-PPQ as first-line therapy in 2008 (1, 2). This combination was associated with a rapid decline in *pfmdr1* copy number (55, 56), which could have resulted from PPQ-mediated selection against multicopy *pfmdr1* and/or selective pressure against multicopy *pfmdr1* parasites that show some loss of fitness and can therefore be outcompeted by single-copy *pfmdr1* parasites (2, 57, 58). In Cambodia, the loss of DHA-PPQ efficacy led to the resumption of artesunate-MFQ use beginning in 2011, and this combination retains good efficacy with *pfmdr1* copy number generally remaining at one (59).

In contrast, the MalariaGEN data illustrate *pfmdr1* amplifications as a rare event in Africa. The substantial use of CQ in the past and the current use of the ACT combination artesunate-ADQ possibly contribute to this fact by selecting for deamplifications (60, 61). Furthermore, although artemether-LMF has been used for many years in Africa (62), the lack of MFQ usage could partially explain the lack of *pfmdr1* amplifications. Nevertheless, it is conceivable that *pfmdr1* amplifications may increase in prevalence in Africa due to the widespread use of artemether-LMF (63, 64). The presence of the 86Y allele in Africa could also contribute to the low prevalence of *pfmdr1* amplifications as this allele is present at a very low frequency worldwide when combined with *pfmdr1* amplifications (6/597 genomes). Another factor could be related to the known fitness cost associated with *pfmdr1* amplifications, which manifests as decreased parasite survival in the absence of drug pressure (58, 65). This feature is of particular relevance in high transmission areas such as in sub-Saharan Africa. An interesting observation is that within the MalariaGEN data, *pfmdr1* amplifications with the haplotype 86Y/184F were not present and despite several attempts to engineer this specific parasite line, we were unsuccessful, possibly related to a genetic background that is detrimental for survival.

Over time in Southeast Asia, we observed a clear selection of the 184F allele in combination with *pfmdr1* amplifications containing the N86 allele. Cambodia was the country with the highest observed selection (25% [3/12] between 2002 and 2007% to 62% [43/69] between 2008 and 2010 and 85% [47/55] between 2011 and 2013) (Fig. 1). It is important to note that at the same time that this takeover of the 184F occurs, the prevalence of *pfmdr1* duplications was actually shrinking due to the switch in first-line treatment to DHA-PPQ. To better understand this selection of the N86/184F haplotype in the context of amplified *pfmdr1*, we generated two edited lines representing the two major geographic variants from Southeast Asia and studied the impact of the specific haplotypes on antimalarial responses. The impact of these polymorphisms on PfMDR1 functional transport was also measured through accumulation of Fluo-4 in the DV of the parasite (Fig. 3). This approach has been previously used for *pfmdr1* variants and revealed an important impact of the C-terminal amino acid 1042 on Fluo-4 transport (22, 23). In these studies, although they used strains encompassing different haplotypes in regard to the *pfmdr1* N-terminal mutations, their direct impact was not evident. In our Fluo-4 accumulation study with the Dd2 strain and isogenic lines differing only in their *pfmdr1* N-terminal residues at positions 86 and 184, we observed that parasites expressing the mutations 86Y and 184F were capable of transporting Fluo-4 into the DV (Fig. 3A). The N86 allele by itself impacted Fluo-4 transport capacity, with significant differences obtained between the parental Dd2 strain and NY^Dd2^ (Fig. 3C). Transport studies in Xenopus oocytes have also provided evidence of the impact of polymorphisms at position 86 on the ability of PfMDR1 to transport different drugs. A single amino acid alteration from N86 to 86Y in PfMDR1 resulted in loss of the ability to transport QN and CQ (21). Consistent with this observation, the presence of the N86 allele induced a significant impact on antimalarial responses (Fig. 4). We were able to confirm the involvement of residue 86, specifically in the context of multicopy *pfmdr1*, in modulating parasite susceptibility to multiple antimalarials. Gene-edited parasites with amplified *pfmdr1* harboring the N86 allele displayed decreased susceptibility to MFQ, LMF, and DHA as well as increased susceptibility to QN, compared to the unedited Dd2 parental line possessing the 86Y allele (Fig. 4). These findings are consistent with our previous allelic exchange work that used ZFN-edited parasites expressing the 86Y variant in the context of single-copy *pfmdr1* (30). This observation explains the selection of the N86 allele after treatment with artemether-LMF (26–28) and corroborates with N86 being a risk factor for recrudescence following treatment with artemether-LMF compared with P. falciparum infections containing 86Y (29).

The *pfmdr1* 184F mutation adds to the role of polymorphisms at position 86 in modulating parasite drug resistance and transport. We found that edited parasites harboring the N86/184F haplotype accumulated more Fluo-4 inside the DV compared to edited parasites with the N86/Y184 haplotype, accompanied by faster kinetics of PfMDR1-mediated transport activity (Fig. 3B). A previous in silico PfMDR1 model proposed that the 184F allele alters the parasite’s response by an allosteric effect on transport kinetics, independent from drug-binding capacity (20). Our gene-edited parasites expressing amplified *pfmdr1* with the N86/184F haplotype displayed a mild but significant decreased susceptibility to LMF (1.7-fold) compared to those harboring the PfMDR1 N86/Y184 haplotype or to the 86Y/Y184-bearing parental Dd2 parasites (Fig. 4). The mild but significant fold difference herein observed was not detected in our previous allelic exchange work for the same haplotype, possibly due to the single *pfmdr1* copy present in the NF10 and KC5 strains precluding sufficient expression to display this phenotype (30). *In vivo* selection of the 184F allele has been reported, typically along with other *pfmdr1* alleles, after artemether-LMF treatment. Most notably, the N86/184F haplotype was selected upon the regular six-dose therapy with artemether-LMF (26, 27). In their analysis of two clinical trials after artemether-LMF treatment, Malmberg et al. demonstrated that recrudescent infections harboring the PfMDR1 N86/184F haplotype, in the context of a single *pfmdr1* copy, were able to endure higher blood LMF concentrations (28). Together with our results that examine the role of copy number, these *in vivo* data suggest that although the *pfmdr1* 184F allele might not be the primary determinant for parasite resistance, it could provide a complementary genetic background that facilitates the acquisition of multidrug-resistant phenotypes, possibly leading to the specific haplotype selection observed in Southeast Asia (Fig. 1).

The interplay between PfMDR1 and PfCRT transporters in modulating parasite susceptibility to antimalarials was also evident in this work (Table 2). Heightened susceptibilities of NF^Dd2^, NY^Dd2^, and Dd2 P. falciparum lines to MFQ, md-ADQ, CQ, and QN were affected by VP as previously observed in other genetic backgrounds (48, 66). The role of PfMDR1 in the parasite’s response to QN remains unclear, but mutant PfCRT is known to contribute to QN resistance (67). Our results with VP also suggest a role for PfCRT. We note that VP increased susceptibility to LMF only in the edited parasites and not in the unedited Dd2 control. Prior data showed selection of the *pfcrt* CQ-sensitive wild-type K76 allele occurs upon artemether-LMF treatment, as confirmed *in vitro* using allelic exchange (68). In the case of LMF, VP might have a greater effect on PfMDR1 in the context of N86, since we observed that VP decreased Fluo-4 accumulation in the parasite’s DV in N86 parasites (Fig. 3D). This observation would explain the increased parasite susceptibility to LMF upon VP exposure.

In the presence of ELC, decreased MFQ IC_50_ values were observed in NF^Dd2^ and NY^Dd2^ edited parasites, reinforcing the hypothesis that not only are *pfmdr1* amplifications associated with MFQ resistance (24, 25, 39) but also that the *pfmdr1* N86 allele is a key mediator of MFQ susceptibility. These findings are in line with the MalariaGEN data showing that almost all parasites that contain *pfmdr1* amplifications are in the context of the *pfmdr1* N86 allele.

In conclusion, our *in vitro* parasite drug susceptibility data using *pfmdr1* gene-edited parasites reinforce the role of both *pfmdr1* CNV and haplotype variation in modulating parasite responses to multiple first-line antimalarial drugs. Our investigation highlights the steady geographic expansion of a parasite population harboring a *pfmdr1* gene amplification with the N86/184F haplotype in Southeast Asia, which we show has a proven higher DV transport efficacy. These data could help tailor and optimize antimalarial drug usage in different areas where malaria is endemic by taking into account the regional prevalence of *pfmdr1* polymorphisms.

## MATERIALS AND METHODS

### Geographical distribution of P. falciparum isolates carrying *pfmdr1* copy number amplifications and the mutations N86Y and Y184F.

Genome analysis used data generated by the Wellcome Sanger Institute as part of the MalariaGEN Plasmodium falciparum Community Project version 6 (https://www.malariagen.net/resource/26) (40). Sample metadata were obtained from https://www.malariagen.net/resource/26 and were combined with the genotype data for *pfmdr1* polymorphisms at codons 86 and 184. *pfmdr1* copy number was determined using a combination of a coverage-based approach and a method based on position and orientation of reads near discovered duplication breakpoints as described elsewhere (41). The map presented in Fig. 1 was created using mapchart.net, geo-referencing the *pfmdr1* gene copy number prevalence in the region.

### Parasite culture.

The Dd2 strain (MRA-156, MR4-Malaria Resources) was maintained at ∼4% hematocrit with human red blood cells in RPMI 1640 medium supplemented with 2 mM l-glutamine, 200 μM hypoxanthine, 0.25 μg/ml gentamicin, 25 mM HEPES, 0.2% NaHCO3, and 0.25% Albumax II (Invitrogen; ThermoFisher Scientific). Red blood cells were isolated either from whole blood or from buffy coat samples provided by healthy donors of all blood types. Parasite cultures were maintained at 37°C under a humidified controlled atmosphere of 5% O2/5% CO2/90% N2. Parasite growth was monitored by inspecting Giemsa-stained blood smears. Parasite synchronization was performed with 5% sorbitol for 15 min at 37°C. To obtain highly synchronous cultures, sorbitol was added to the culture to eliminate trophozoites. This was repeated 20 h and 44 h after the initial treatment to obtain 4-h postinvasion ring stages that then mature into trophozoites, which were used to measure Fluo-4 fluorescence.

### P. falciparum transfections.

The previously designed plasmids p*mdr1*^NF^ and p*mdr1*^NY^ (30) were used to transfect Dd2 parasites. Briefly, we introduced the desired mutations into the endogenous *pfmdr1* locus of Dd2 parasites using ZFN-mediated genome editing. These customized ZFNs bind on opposite strands of *pfmdr1*, producing a double-stranded break 42 bp downstream of the start codon. DNA repair proceeds via homologous recombination, as P. falciparum lacks the nonhomologous end-joining pathway (69). Our homology-driven repair template consisted of a 2.4-kb *pfmdr1* fragment that encompassed codons 86 and 184 (30).

Ring-stage cultures at 5% parasitemia were electroporated with ∼50 μg of plasmid DNA diluted in Cytomix (120 mM KCl, 0.15 mM CaCl_2_, 2 mM EGTA, 5 mM MgCl_2_, 10 mM K_2_HPO_4_/KH_2_PO_4_, 25 mM HEPES [pH 7.6]). The plasmids express the human dihydrofolate reductase (hDHFR) selectable marker, which confers resistance to the antifolate drug WR99210. To allow transient expression of the ZFNs, WR99210 selection was applied to transfected cultures beginning 24 h postelectroporation and maintained for 6 days to select for edited parasites. Parasites were visible microscopically 6 weeks postelectroporation and screened for editing events via blood PCR using the primers p3 and p4. Positive bulk cultures were cloned by limiting dilution in 96-well plates. After 15 days, cloning plates were screened and parasite-positive wells were screened for ZFN-mediated editing by blood PCR amplification using a supreme NZYTaq II 2× Green Master Mix (NZYTech, Lisbon, Portugal) and the p3 and p4 primers (see Table S2 in the supplemental material). The donor sequence includes silent mutations that create a SmlI restriction site, which was used to screen the PCR products by restriction fragment length polymorphism (RFLP) analysis (a 1.3-kb band obtained with primers P3 plus P4 [Table S2] that forms a doublet at 667/669 bp with SmlI in edited parasites). The BstEII restriction site is present only in the nonedited genomic *pfmdr1* sequence and was therefore used as a control in the PCR-RFLP assay (Fig. 2B). Editing events at codons 86 and 184 were confirmed by Sanger sequencing using the p5 primer (Table S2). Successfully edited parasite lines (NF^Dd2^ and NY^Dd2^) were expanded and cryopreserved.

10.1128/mBio.02093-20.4 TABLE S2 List of primers used in this study. Primers p1 and p2 were used to perform site-directed mutagenesis at amino acid positions 86 and 184 of *pfmdr1* to obtain the desired sequence. Primers p3 and p4 were used to detect the *pfmdr1*-modified parasites. Primers *pfmdr1* and the reference gene β-tubulin were used to determine copy number variation at the *pfmdr1* locus. Download Table S2, XLSX file, 0.01 MB. Copyright © 2020 Calçada et al. 2020 Calçada et al. This content is distributed under the terms of the Creative Commons Attribution 4.0 International license.

In this work, we have compared the edited NF^Dd2^ and NY^Dd2^ lines to an unedited Dd2 parental control harboring the 86Y/Y184 haplotype. An edited Dd2 control was not performed in this study, since the plasmids used herein to edit Dd2 parasites were the same as those used previously by our group (30). In that study, we demonstrated that gene-edited control parasites carrying only silent ZFN binding site mutations did not alter the parasite’s response to antimalarial drugs compared with unedited parental lines.

### Copy number analysis of *pfmdr1*.

Genomic DNA from parental control Dd2 and *pfmdr1*-edited parasites (NY^Dd2^ and NF^Dd2^) were extracted using the NZY Blood gDNA isolation kit (NZYTech, Lisbon, Portugal) with RNase treatment. Copy numbers of the *pfmdr1* gene were determined using a NZYSpeedy Green Master Mix (2×) (NZYTech, Lisbon, Portugal). The amplification reactions were carried out in 20-μl volumes in a 96-well plate using 10 ng of genomic DNA, 400 nM each forward and reverse primer, and the master mix. The P. falciparum β-tubulin gene was used as the housekeeping control gene. The *pfmdr1* and pfβ-tubulin forward and reverse primers (Table S2) were previously designed (70). For each run, 3D7 was used as a single-copy-number control for *pfmdr1*. Thermal cycling was performed at 98°C for 3 min, followed by 44 cycles with 1 cycle consisting of 98°C for 15 s, 59°C for 30 s, and 72°C for 25 s using the Bio-Rad CFX96 real-time system C1000 thermal cycler. Results were analyzed by the 2 − ΔΔCt (threshold cycle) method of relative quantification. The ΔΔCt calculation was used as follows ΔΔ*Ct* = (*Ct* of *pfmdr1* – *Ct* of Pfβ-tubulin)χ – (*Ct* of *pfmdr1* – *Ct* of Pfβ-tubulin)y, where χ is sample and *y* is P. falciparum 3D7 strain. The average gene copy number was calculated from three biological replicates, each with technical replicates run in duplicate.

### Live-cell imaging of Fluo-4 accumulation in the DV.

Fluorescence microscopy was used to evaluate the accumulation site of Fluo-4 AM (Invitrogen; ThermoFisher Scientific) in different parasite strains in trophozoite stages. Synchronized trophozoite cultures, at ∼2% parasitemia and 2% hematocrit, were washed with RPMI 1640 medium and loaded with 5 μM Fluo-4 diluted in RPMI 1640 medium. Parasites were incubated for 240 min at 37°C in a humidified controlled atmosphere of 5% O2/5% CO2/90% N2. After 240 min, the parasites were washed twice with 1× phosphate-buffered saline (PBS) and transferred onto a slide. Fluorescence microscopy was performed using an Olympus BX61 microscope equipped with a visible light laser, and the images were recorded with a digital camera (DP70). The parasites incubated with Fluo-4 were excited at 488 nm with the emission in the green channel (505-nm filter), and an exposure time of 457 ms was applied to obtain the images. Single images were obtained using a 100× objective lens. Regions of interest within the infected RBC, including the parasite cytosol and the parasite DV, were recorded with Cell̂P software (Electro optics, UK) and the image overlays were obtained using Image J software version 1.52a.

### Quantification of Fluo-4 accumulation by flow cytometry.

Flow cytometry was used to quantify Fluo-4 accumulation in NF^Dd2^, NY^Dd2^, Dd2, and W2 parasites. Parasite labeling and evaluation of Fluo-4 accumulation were performed as described previously (22) with minor modifications. Briefly, synchronized trophozoites were incubated with 5 μM Fluo-4 AM (Invitrogen; ThermoFisher Scientific) for 240 min at 37°C in an airtight environment flushed with 5% O2/5% CO2/90% N2. The level of fluorescence in infected RBCs was recorded by flow cytometry every 15 min until 120 min, and every 30 min thereafter up to a total incubation period of 240 min (see Fig. S2 in the supplemental material). This assay was followed by live-cell imaging at all time points described above to visualize when the Fluo-4 probe accumulated in the parasite DV (Fig. S2). Fluo-4 accumulation in the DV, presented in Fig. 3, was confirmed by fluorescence microscopy before flow cytometry. Thirty minutes before the incubation ended, parasites were loaded with 1.6 μM Mitotracker Deep Red FM (Invitrogen; ThermoFisher Scientific). For flow cytometry, labeled parasites were excited at 488 nm and 633 nm to detect Fluo-4 and Mitotracker Deep Red FM, respectively. Forward (FSC) and side scatterplots (SSC) were used to define the RBC populations followed by gating double-positive populations for Fluo-4 and Mitotracker Deep Red FM. Approximately 200,000 events were read per experimental condition, and the mean Fluo-4 intensity was calculated for each parasite strain. Due to intrinsic variability observed in the different replicate assays, the fluorescence intensity of NF^Dd2^, NY^Dd2^, and W2 was normalized to the Dd2 parental control (i.e., Dd2 corresponding to 100%; indicated in the graph as the baseline [*y* = 0]). Values below zero indicate a decrease in the percentage of accumulated fluorescence compared with Dd2 parasites, whereas values above zero indicate an increase.

Fluo-4 accumulation in NF^Dd2^, NY^Dd2^, and Dd2 parasites was also measured in the presence or absence of ELC, an inhibitor of P-type ATPase transporters, including PfMDR1 (and others, e.g., ABCI3), or VP, a calcium channel blocker that functions as a CQ resistance reversal agent. Briefly, synchronized trophozoites were washed with RPMI 1640 medium and preincubated for 10 min with ELC (0.1 μM) or VP (0.8 μM). Sample preparation and flow cytometry were performed as described above. The fluorescence intensities of NF^Dd2^, NY^Dd2^, and Dd2 in the presence of VP or ELC were normalized to the respective untreated controls (indicated in the graph as the baseline [*y* = 0]). Values below zero indicate a decrease in the percentage of accumulated fluorescence compared with untreated controls, while values above zero indicate an increase.

### *In vitro* antimalarial drug assays.

Drug susceptibility assays using CQ, DHA, MFQ, QN, LMF, md-ADQ, PPQ, and PND in the absence or presence of ELC (0.1 μM) or VP (0.8 μM) (48) were run for the edited parasite lines NF^Dd2^ and NY^Dd2^, the parental control strain Dd2, and the W2 strain. These assays were performed using a published flow cytometry-based method (30). Briefly, synchronized ring-stage parasites at 0.2% starting parasitemia and 1% hematocrit were incubated in the presence of different concentrations of drug (across a dilution range of twofold; 12-point dilution series). After 72 h of incubation at 37°C, samples were stained with 1.6 μM Mitotracker Deep Red FM (Invitrogen; ThermoFisher Scientific) and 2× SYBR green (for DNA staining) (Invitrogen; ThermoFisher Scientific) in 1× PBS for 30 min and analyzed by flow cytometry to determine the parasite growth rates. Approximately 100,000 events were captured per well. *In vitro* IC_50_ values were calculated using nonlinear regression analysis performed with GraphPad Prism 6 software.

### Statistical analysis.

Nonparametric, two-tailed Mann-Whitney U tests were used to assess IC_50_ antimalarial responses in the presence or absence of VP or ELC and Fluo-4 accumulation differences between the NF^Dd2^ and NY^Dd2^, Dd2, and W2 parasite strains (normal distribution not assumed; performed with GraphPad Prism Software).
