# Supplementary figures and images for: Expansion of a Specific Plasmodium falciparum PfMDR1 Haplotype in Southeast Asia with Increased Substrate Transport
Source: mBio. 2020 Dec 1;11(6):e02093-20. doi: 10.1128/mBio.02093-20 (PMC7733942; doi:10.1128/mBio.02093-20)

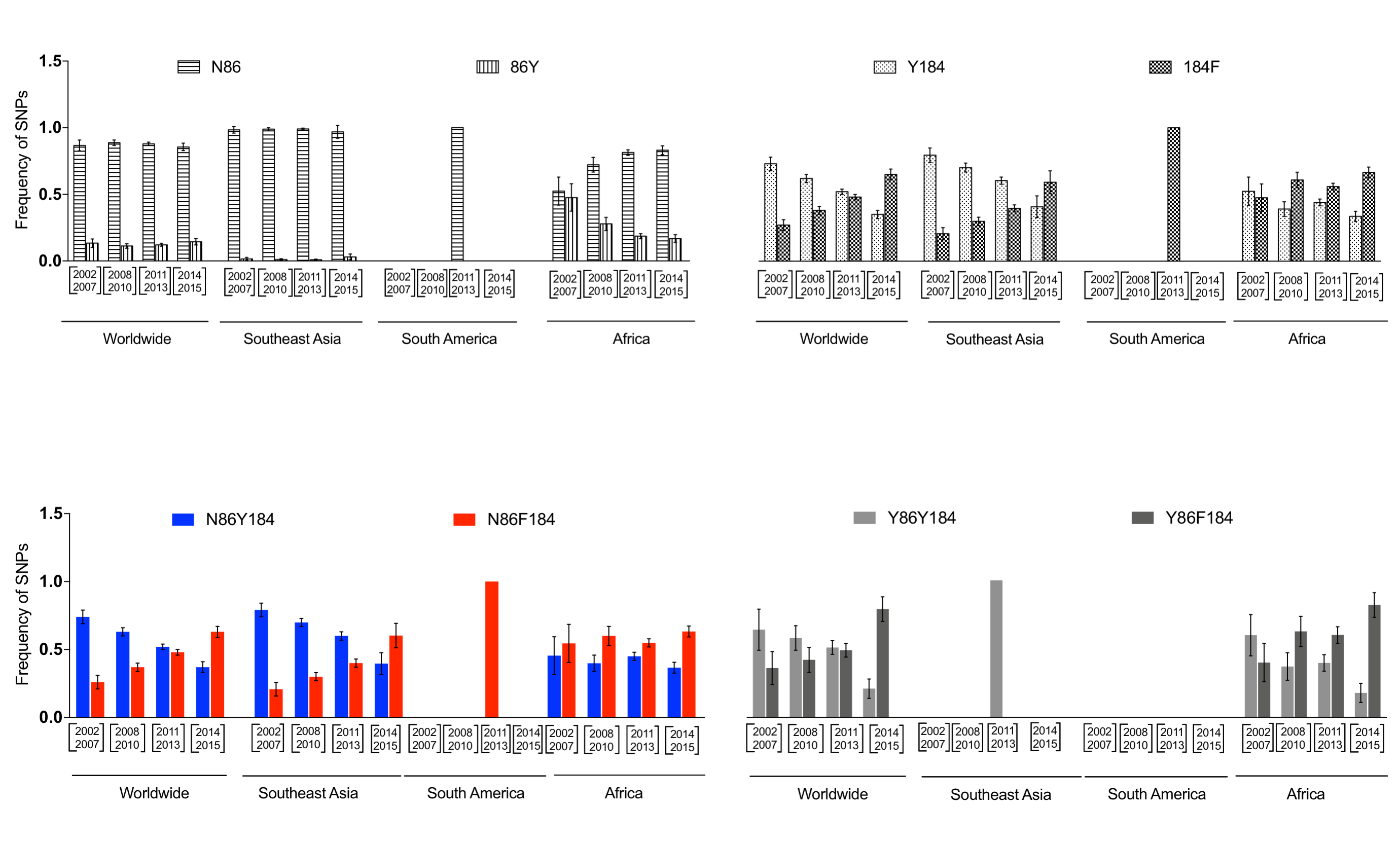

Supplement: FIG S1 [file mBio.02093-20-sf001.tif]

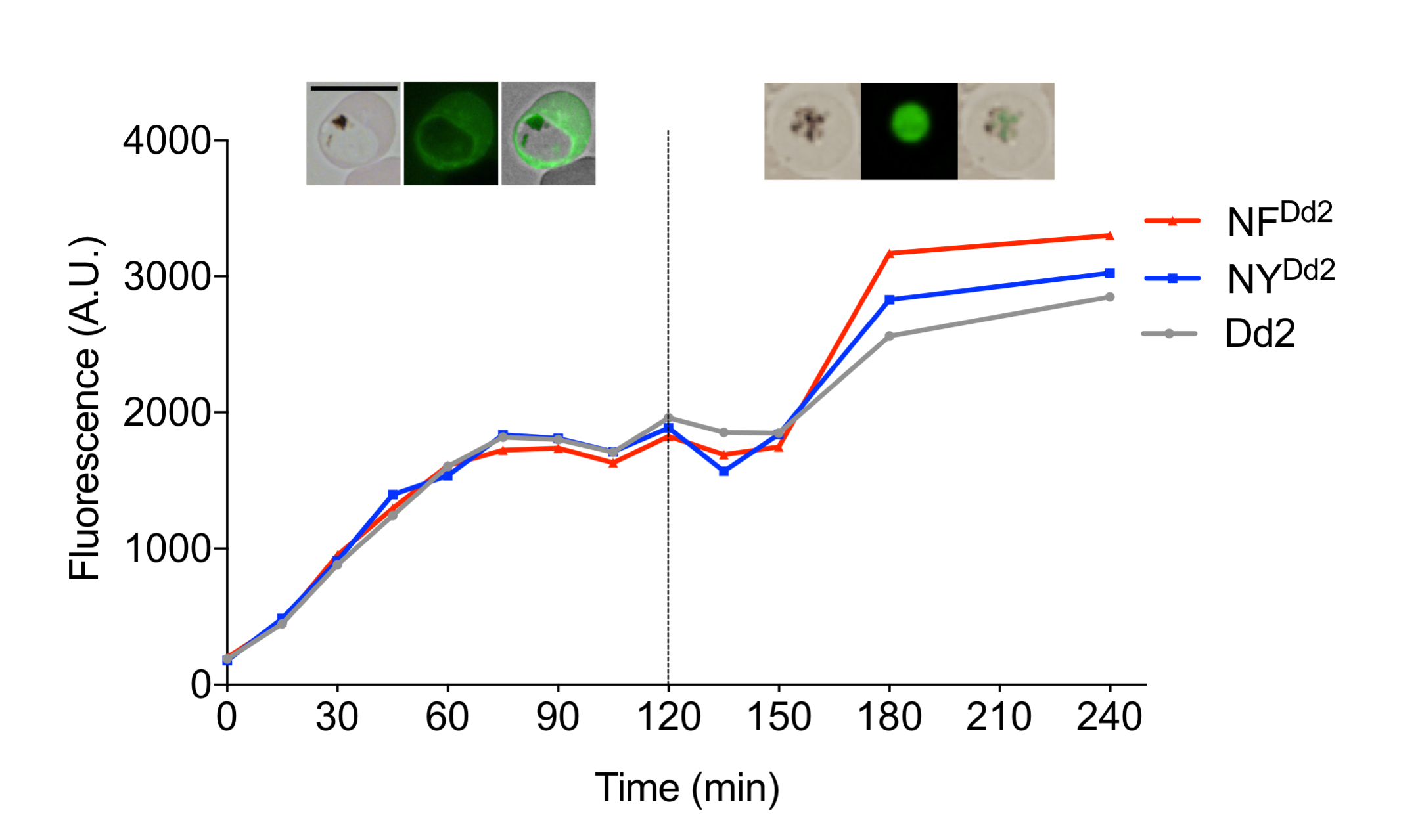

Supplement: FIG S2 [file mBio.02093-20-sf002.tif]
